# Early sedation and clinical outcomes of mechanically ventilated patients: a prospective multicenter cohort study

**DOI:** 10.1186/cc13995

**Published:** 2014-07-21

**Authors:** Lilian Maria Sobreira Tanaka, Luciano Cesar Pontes Azevedo, Marcelo Park, Guilherme Schettino, Antonio Paulo Nassar, Alvaro Réa-Neto, Luana Tannous, Vicente Ces de Souza-Dantas, André Torelly, Thiago Lisboa, Claudio Piras, Frederico Bruzzi Carvalho, Marcelo de Oliveira Maia, Fabio Poianas Giannini, Flavia Ribeiro Machado, Felipe Dal-Pizzol, Alexandre Guilherme Ribeiro de Carvalho, Ronaldo Batista dos Santos, Paulo Fernando Guimarães Morando Marzocchi Tierno, Marcio Soares, Jorge Ibrain Figueira Salluh

**Affiliations:** 1 Hospital Copa D’Or, Rua Figueiredo de Magalhães 875, Rio de Janeiro 22031-010, Brazil; 2 Research and Education Institute, Hospital Sírio-Libanês, Rua Cel. Nicolau dos Santos 69, São Paulo 01308-060, Brazil; 3 ICU, Emergency Medicine Department, Hospital das Clinicas da Faculdade de Medicina da Universidade de São Paulo, Av. Eneas Carvalho Aguiar 255, São Paulo 05403-000, Brazil; 4 ICU, Hospital São Camilo Pompeia, Av. Pompeia 1178, São Paulo 05022-000, Brazil; 5 CEPETI – Centro de Estudos e Pesquisas em Terapia Intensiva, Rua Monte Castelo 366, Curitiba 82530-200, Brazil; 6 ICU, Instituto Nacional de Câncer – Hospital do Câncer I, Praça Cruz Vermelha 23, Rio de Janeiro 20230-130, Brazil; 7 ICU, Hospital Pasteur, Av. Amaro Cavalcanti 495, Rio de Janeiro 20735-040, Brazil; 8 Rede Institucional de Pesquisa e Inovação em Medicina Intensiva (RIPIMI), Irmandade da Santa Casa de Misericórdia de Porto Alegre, Rua Professor Annes Dias 285 – Centro Histórico, Porto Alegre 90020-090, Brazil; 9 ICU, Vitoria Apart Hospital, Rodovia BR-101 Norte, Km 2, 38 Boa Vista II, Serra, ES 29161-900, Brazil; 10 ICU, Hospital Mater Dei, Rua Gonçalves Dias 2700 (Bloco I), Belo Horizonte 30140-093, Brazil; 11 ICU, Hospital Santa Luzia, SHLS 716 – Conjunto E, Brasília 70390-902, Brazil; 12 ICU, Hospital São Luiz, Unidade Itaim, Rua Doutor Alceu de Campos Rodrigues 95, São Paulo 04544-000, Brazil; 13 ICU, Anesthesiology, Pain and Intensive Care Department, Universidade Federal de São Paulo, Rua Napoleão de Barros, 715 6° andar, São Paulo 04024-900, Brazil; 14 ICU, Hospital São José Criciúma, Rua Coronel Pedro Benedet 630, Criciúma 88801-250, Brazil; 15 ICU, UDI Hospital, Av Professor Carlos Cunha 2000, São Luis 65076-820, Brazil; 16 ICU, Hospital Universitário da Universidade de São Paulo, Av Prof. Lineu Prestes 2565 – Cidade Universitária, São Paulo 05508-000, Brazil; 17 ICU, Surgical Emergency Department, Hospital das Clinicas da Faculdade de Medicina da Universidade de São Paulo, Av. Eneas Carvalho Aguiar 255, São Paulo 05403-000, Brazil; 18 IDOR – D’Or Institute for Research and Education, Rua Diniz Cordeiro 30, Rio de Janeiro 22281-100, Brazil; 19 Postgraduate Program, Instituto Nacional de Câncer, 10° Andar, Praça Cruz Vermelha 23, Rio de Janeiro 20230-130, Brazil

## Abstract

**Introduction:**

Sedation overuse is frequent and possibly associated with poor outcomes in the intensive care unit (ICU) patients. However, the association of early oversedation with clinical outcomes has not been thoroughly evaluated. The aim of this study was to assess the association of early sedation strategies with outcomes of critically ill adult patients under mechanical ventilation (MV).

**Methods:**

A secondary analysis of a multicenter prospective cohort conducted in 45 Brazilian ICUs, including adult patients requiring ventilatory support and sedation in the first 48 hours of ICU admissions, was performed. Sedation depth was evaluated after 48 hours of MV. Multivariate analysis was used to identify variables associated with hospital mortality.

**Results:**

A total of 322 patients were evaluated. Overall, ICU and hospital mortality rates were 30.4% and 38.8%, respectively. Deep sedation was observed in 113 patients (35.1%). Longer duration of ventilatory support was observed (7 (4 to 10) versus 5 (3 to 9) days, *P* = 0.041) and more tracheostomies were performed in the deep sedation group (38.9% versus 22%, *P* = 0.001) despite similar PaO_2_/FiO_2_ ratios and acute respiratory distress syndrome (ARDS) severity. In a multivariate analysis, age (Odds Ratio (OR) 1.02; 95% confidence interval (CI) 1.00 to 1.03), Charlson Comorbidity Index >2 (OR 2.06; 95% CI, 1.44 to 2.94), Simplified Acute Physiology Score 3 (SAPS 3) score (OR 1.02; CI 95%, 1.00 to 1.04), severe ARDS (OR 1.44; CI 95%, 1.09 to 1.91) and deep sedation (OR 2.36; CI 95%, 1.31 to 4.25) were independently associated with increased hospital mortality.

**Conclusions:**

Early deep sedation is associated with adverse outcomes and constitutes an independent predictor of hospital mortality in mechanically ventilated patients.

## Introduction

Sedation is an important component of care for patients under mechanical ventilation (MV) in the ICU. Significant distress is related to MV itself or to routine procedural interventions [1] and minimizing pain, anxiety and distress is a major recommendation in recent guidelines [2]. Pain and anxiety control is usually obtained with analgesics and sedatives that ensure comfort, improve synchrony with the ventilator and decrease work of breathing [3]. Some studies, however, have shown that oversedation is associated with poor outcomes, including delirium, prolonged MV, ventilator-associated pneumonia, long ICU and hospital length of stay [3-6], posttraumatic stress disorder [7] and cognitive impairment [8,9] as well as increased costs [10-12]. Nevertheless, the issue of early sedation has seldom been evaluated, especially in randomized controlled studies.

Despite the current recommendations [2], there is still a significant gap between evidence from recent trials and implementation in clinical practice [2,6,13,14]. Moreover, to date no large randomized controlled trials of sedation strategies used mortality as the primary outcome. In addition, clinical trials of sedation have until now enrolled patients mostly after 24 to 48 hours following initiation of MV, resulting in inadequate assessment of early sedation practice and its association with clinically relevant outcomes [15-17].

The aim of this study is thus to describe the association of early sedation strategies (sedation depth and sedative choice) with clinical outcomes of mechanically ventilated adult ICU patients, with hospital mortality as the primary outcome.

## Materials and methods

### Study design, patient selection and definitions

This study was a secondary analysis of a multicenter prospective cohort conducted in 45 Brazilian ICUs (from the Brazilian Research in Intensive Care Network) from 12 states between 1 June 2011 and 31 July 2011 [18]. Additional file 1 presents the participating ICUs in more detail. In the present study, adult patients (≥18 years old) requiring invasive ventilatory support in the first 48 hours of ICU admission using sedatives on the second day of MV were included.

We excluded patients submitted exclusively to noninvasive MV and those presenting with primary neurological disorders. We also excluded those with missing data regarding sedation depth on the second day of MV. Demographic, clinical and laboratory data were collected during the ICU stay and included cause of respiratory failure, chronic health status, Charlson Comorbidity Index [19], Simplified Acute Physiology Score 3 (SAPS3) [20] and the Sequential Organ Failure Assessment (SOFA) score [21]. Data regarding the need for vasopressors, tracheostomy and renal replacement therapy were also reported. Sepsis diagnosis was made based on current definitions [22]. A patient was considered to have an infection when presenting with suggestive clinical, laboratory, radiographic and microbiological findings that justified the administration of antibiotics (excluding prophylaxis) [23]. Acute respiratory distress syndrome (ARDS) was defined and classified according to the Berlin definition [24].

As the primary study was not designed to evaluate sedation strategies [18], the Glasgow Coma Scale (GCS) – originally reported for SOFA score calculation – was used as a surrogate for sedation depth. Light sedation was defined as GCS ≥9 whereas those patients with GCS <9 were considered deeply sedated. Reliability of GCS as a surrogate for the Richmond Agitation–Sedation Scale (RASS) as well as selection of GCS = 9 as the cutoff value were based on the study by Ely and colleagues that demonstrated an excellent correlation (*r* = 0.91, *P* <0.001) between the GCS and RASS, where GCS = 9 would be equivalent to RASS = -2 [25].

Our primary hypothesis was that early sedation strategies, namely sedation depth, could be associated with clinical outcomes including hospital mortality, so we chose to evaluate the sedation level on the second day of MV.

The study was approved by the institutional review board of Hospital Sírio-Libanês at the coordinating center (Comitê de Ética em Pesquisa; approval number HSL 2010/51) and subsequently by local review boards. Additional file 1 describes the local review boards by participating hospital. Due to the observational nature of the study, the following institutions waived the need for informed consent: Comitê de Ética em Pesquisa – Hospital São Camilo; Comitê de Ética em Pesquisa – Hospital Sírio-Libanês; Comissão de Ética para Análise de Projetos de Pesquisa – HCFMUSP; Comitê de Ética em Pesquisa – Hospital das Clinicas de Niteroi; Comitê de Ética em Pesquisa – Hcor; Comitê de Ética em Pesquisa – Hospital Israelita Albert Einstein; Comitê de Ética em Pesquisa – HCFMRP – USP; Comitê de ética do Hospital Sao Jose; Comitê de Ética em Pesquisa do Vitória Apart Hospital; Comitê de Ética em Pesquisa do Hospital Madre Teresa; Comitê de Ética em Pesquisa do Hospital da Mulher Heloneida Studart; Comitê de Ética em Pesquisa – Hospital Moinhos de Vento; Comitê de Ética em Pesquisa do Hospital Mater Dei; Comitê de Ética em pesquisa – Hospital Mario Lioni; Comitê de Ética e Pesquisa do Hospital Espanhol; Comitê de Ética em Pesquisa – Instituto de Pesquisa Clínica Evandro Chagas, Fundação Oswaldo Cruz; Comitê de Ética em Pesquisa – UNIFESP/EPM; Comitê de Ética em Pesquisa – Hospital Copa D’or; Comitê de Ética em Pesquisa – Beneficiência Médica Brasileira S/A Hospital São Luiz; Comitê de Ética em Pesquisa do Hospital do Trabalhador; Comitê de Ética em Pesquisa da Fundação de Medicina Tropical do Tocantins; and Comitê de Ética em Pesquisa – FMUSP.

### Statistical analysis

Continuous variables are reported as the median (25 to 75% interquartile range). Univariate and multivariate analyses using a binary logistic regression were used to identify factors associated with the dependent variable (hospital mortality). Variables yielding *P* <0.2 by univariate analysis, or those considered clinically relevant despite *P* values, were entered into the multivariate analysis to estimate the independent association of each covariate with the dependent variable. Results are presented as the odds ratio with 95% confidence interval. A Kaplan–Meier curve with log-rank test was used to compare patients with light versus deep sedation for hospital mortality (censored at day 30). Two-tailed *P* <0.05 was considered statistically significant. All statistical tests were carried out using the commercial SPSS19.0 package for Windows (SPSS Inc., Chicago, IL, USA).

## Results

### Characteristics of the study population

**Figure 1:**
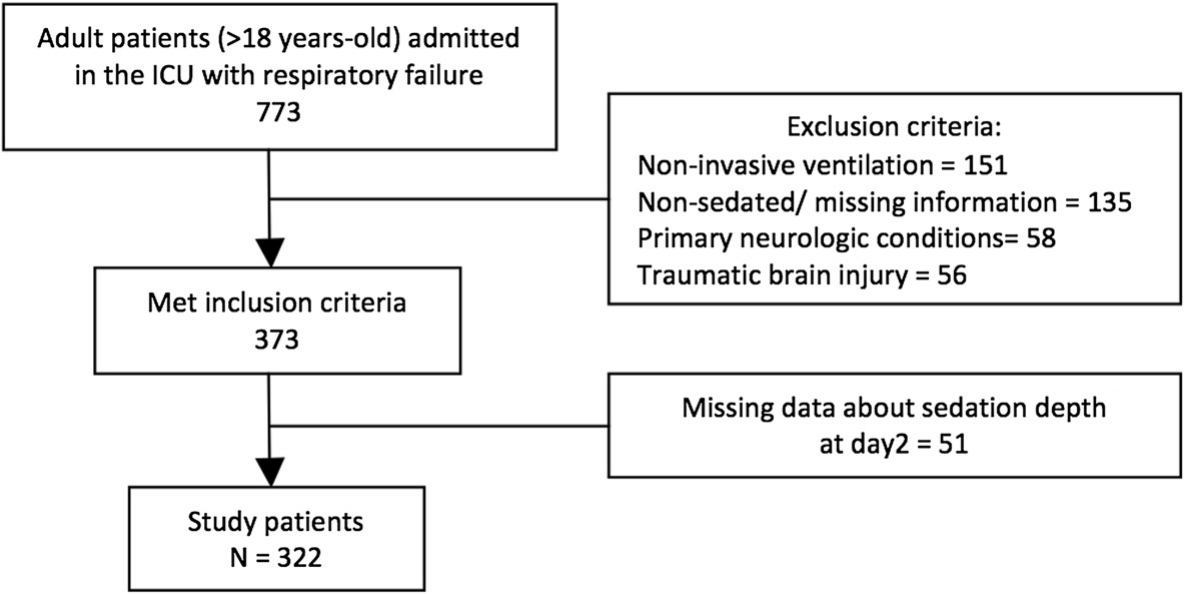
Study flow diagram.

From 773 patients derived from the original cohort, a total of 322 patients fulfilled the eligibility criteria (Figure 1). Patients excluded for missing data about sedation depth on day 2 (*n* = 51) were comparable with the included population regarding demographic data, comorbidities, physiologic and disease severity variables and outcomes (data not shown). The main patient characteristics are presented in Table 1. Median (25 to 75% interquartile range) age was 59 (41 to 74) years. The median SAPS3 was 61 (50 to 72) and SOFA score was 8 (6 to 11) on the first day in the ICU. Overall ICU and hospital mortality rates were 30.4% and 38.8% respectively. When comparing nonsurvivor and survivor subgroups, we observed that nonsurvivors showed higher median age (68 (52 to 77) years vs. 53 (34 to 69) years, *P* <0.001), higher SAPS3 and SOFA score (68 (57 to 79) vs. 57 (48 to 67), *P* < 0.001 and 10 (7 to 12) vs. 8 (5 to 10), *P* <0.001) and higher percentage of patients with a Charlson Comorbidity Index >2 (37.6% vs. 16.2%, *P* <0.001). Those who died during hospital admission also had a higher frequency of moderate/severe ARDS (24% vs. 9.6%, *P* = 0.004), need for vasopressors (85.6% vs.71.1%, *P* = 0.003) and renal replacement therapy (33.6% vs.13.7%, *P* <0.001).

**Table 1:** General characteristics of study patients according to survival status

**Characteristic**	**Survivors (*n *** **=197)**	**Nonsurvivors (*n *** **=125)**	***P *** **value**^**a**^
Age (years)	53 (34 to 69)	68 (52 to 77)	<0.001
Male gender	114 (58)	73 (58)	0.925
Admission SAPS3 (points)	57 (48 to 67)	68 (57 to 79)	<0.001
SOFA score day 1 (points)	8 (5 to 10)	10 (7 to 12)	<0.001
Charlson comorbidity index			
0	106 (53.8)	30 (24)	
1 to 2	59 (29.9)	48 (38.4)	<0.001
> 2	32 (16.2)	47 (37.6)	
Admission source			0.038
Ward	52 (26.4)	49 (39.2)	
Emergency room	79 (40.1)	46 (36.8)	
Operation room	66 (33.5)	30 (24)	
Cause of respiratory failure			
Pneumonia	66 (33.5)	39 (31.2)	0.668
Nonpulmonary sepsis	30 (15.2)	15 (12)	0.415
Asthma/COPD	23 (11.7)	7 (5.6)	0.068
Cardiogenic pulmonary edema	11 (5.6)	9 (7.2)	0.558
Extracranial trauma	11 (5.6)	12 (9.6)	0.173
Hypovolemic/cardiogenic shock	10 (5.1)	8 (6.4)	0.614
Aspirative syndromes	10 (5.1)	3 (2.4)	0.234
Pulmonary embolism	4 (2)	5 (4)	0.296
Others	32 (16.2)	27 (21.6)	0.226
Comorbidities			
Hypertension	63 (32)	58 (46.4)	0.008
Diabetes	30 (15.2)	42 (33.6)	<0.001
Heart failure	12 (6.1)	14 (11.2)	0.101
COPD	12 (6.1)	12 (9.6)	0.243
Chronic renal failure	11 (5.6)	23 (18.4)	<0.001
Respiratory data			
ARDS mild	29 (14.7)	21 (16.8)	0.004
ARDS moderate/severe	19 (9.6)	30 (24)	
PO_2_/FiO_2_ ratio	287 (220 to 350)	252 (184 to 328)	0.019
Tracheostomy	54 (27.4)	36 (28.8)	0.817
Extubation failure	23 (11.7)	19 (15.2)	0.182
Length of ventilatory support (days)	5 (3 to 8)	6 (3 to 11)	0.260
ICU support			
Renal replacement therapy	27 (13.7)	42 (33.6)	<0.001
Vasopressors	140 (71.1)	107 (85.6)	0.003
Outcomes			
ICU length of stay (days)	11 (7 to 19)	11 (5 to 18.5)	0.389
Hospital length of stay (days)	23 (14 to 36)	14.5 (7.3 to 27)	<0.001
Sedation depth			
Light sedation	136 (69.0)	73 (58.4)	0.051
Deep sedation	61 (31.0)	52 (41.6)	

Data presented as median (25 to 75% interquartile range) or *n* (%). ARDS, acute respiratory distress syndrome; COPD, chronic obstructive pulmonary disease; PO_2_/FiO_2_, arterial partial pressure of oxygen/fraction of inspired oxygen; SAPS3, Simplified Acute Physiology Score 3; SOFA, Sequential Organ Failure Assessment. ^a^*P* value of the comparison between survivors and nonsurvivors.

### Sedative choice and sedation depth

The most frequently used sedatives were the association of midazolam and fentanyl (39.4%) or of propofol and fentanyl (14%) and the use of fentanyl (12.4%) or midazolam (8.1%) as single drugs. Sedative drug type was also associated with sedation levels: the sole administration of fentanyl and dexmedetomidine was more frequent in lightly sedated patients (15.3% vs. 7.1% for fentanyl, *P* = 0.033; 8.6% vs. 0.9% for dexmedetomidine, *P* = 0.005).

**Figure 2:**
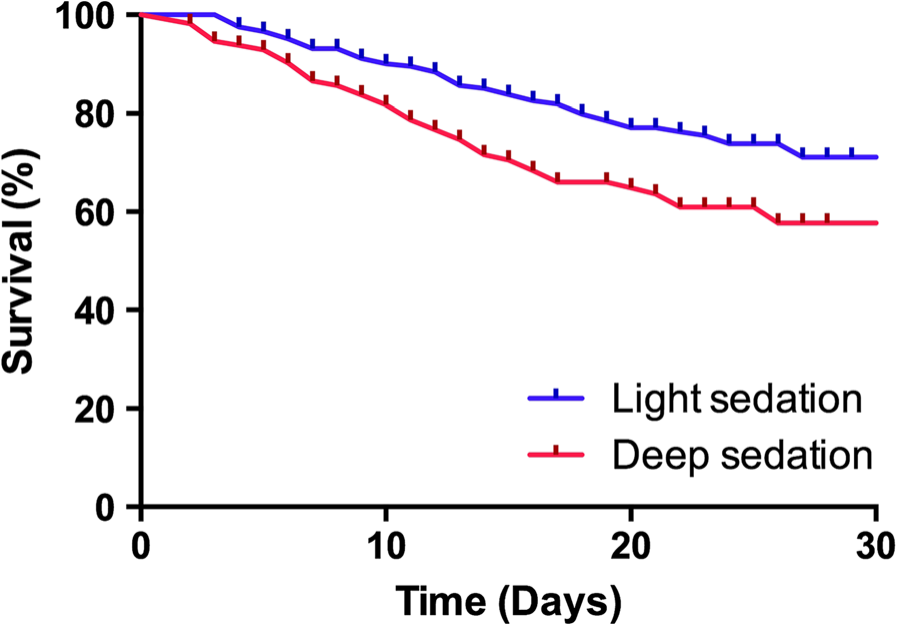
**Kaplan–Meier analysis depicting the impact of sedation depth on hospital mortality.** Blue line, patients with light sedation at day 2; red line, patients with deep sedation at day 2. *P* = 0.051.

Deep sedation was observed in 113 patients (35.1%) and was associated with a higher overall disease severity, as demonstrated by higher median SAPS 3 (65 (54 to 78) for deep sedation vs. 59 (48 to 70) for light sedation, *P* = 0.001) (Table 2). Moreover, longer duration of ventilatory support was observed (7 (4 to 10) vs. 5 (3 to 9) days, *P* = 0.041) and more tracheostomies were performed in the deep sedation group (38.9% vs. 22%, *P* = 0.001) despite similar arterial partial pressure of oxygen/fraction of inspired oxygen ratios and ARDS severity. Finally, trends to increased ICU and hospital mortality (37.2% vs. 26.8%, *P* = 0.054 and 46% vs. 34.9%, *P* = 0.051 respectively) were also associated with deep sedation (Table 3). A survival curve comparing patients with deep and light sedation is shown in Figure 2.

**Table 2:** General characteristics of study patients according to sedation depth

**Characteristic**	**All patients (*n*** **= 322)**	**Light sedation (*n*** **= 209)**	**Deep sedation (*n*** **= 113)**	***P *** **value**^**a**^
Age (years)	59 (40.8 to 74)	61 (44 to 74.5)	55 (33.5 to 73)	0.068
Male gender	187 (58)	108 (52)	79 (70)	0.002
Charlson comorbidity index				
0	136 (42.2)	72 (34.4)	64 (56.7)	<0.001
1 to 2	107 (33.2)	75 (35.9)	32 (28.3)	
> 2	79 (24.5)	62 (29.7)	17 (15)	
Comorbidities				
Hypertension	121 (37.7)	88 (42.1)	33 (29.2)	0.026
Diabetes	72 (22.4)	50 (25.4)	22 (17.6)	0.381
Heart failure	26 (8.1)	21 (10.7)	5 (14.7)	0.077
COPD	24 (7.5)	21 (10)	3 (2.7)	0.016
Chronic renal failure	34 (10.6)	24 (12.2)	10 (8)	0.463
Admission source				0.103
Ward	101 (31.4)	74 (35.4)	27 (23.9)	
Emergency room	125 (38.8)	77 (36.8)	48 (42.5)	
Operation room	96 (29.8)	58 (27.8)	38 (33.6)	
Admission scores (points)				
Admission SAPS3	61 (50 to 72)	59 (48 to 70)	65 (54 to 78)	0.001
SOFA score day 1	8 (6 to 11)	8 (5 to 11)	9 (7 to 11)	0.001
Cause of respiratory failure				
Pneumonia	105 (32.6)	69 (33)	36 (31.9)	0.833
Nonpulmonary sepsis	45 (14)	30 (14.4)	15 (13.3)	0.790
Asthma/COPD	30 (9.3)	19 (9.1)	11 (9.7)	0.850
Cardiogenic pulmonary edema	20 (6.2)	15 (7.2)	5 (4.4)	0.329
Extracranial trauma	23 (7.1)	13 (6.2)	10 (8.8)	0.382
Hypovolemic/cardiogenic shock	18 (5.6)	12 (5.7)	6 (5.3)	0.872
Aspirative syndromes	13 (4)	9 (4.3)	4 (3.5)	0.739
Pulmonary embolism	9 (2.8)	6 (2.9)	3 (2.7)	0.911
Others	59 (18.3)	36 (17.2)	23 (20.4)	0.488

Data presented as median (25 to 75% interquartile range) or *n* (%). COPD, chronic obstructive pulmonary disease; SAPS3, Simplified Acute Physiology Score 3; SOFA, Sequential Organ Failure Assessment. ^a^*P* value of the comparison between light and deep sedation.

**Table 3:** Clinical outcomes according to sedation depth

**Clinical outcomes**	**All patients (*n*** **= 322)**	**Light sedation (*n*** **= 209)**	**Deep sedation (*n*** **= 113)**	***P *** **value**^**a**^
Respiratory data				
ARDS mild	50 (15.5)	35 (16.7)	15 (13.3)	0.521
ARDS moderate/severe	49 (15.2)	35 (16.7)	14 (12.4)	
PO_2_/FiO_2_ ratio	278 (208 to 339)	268 (208 to 329)	282 (208 to 353)	0.486
Tracheostomy	90 (28.3)	46 (22)	44 (38.9)	0.001
Extubation failure	42 (13.8)	32 (15.3)	10 (8.8)	0.119
Length of ventilatory support (days)	5 (3 to 9)	5 (3 to 9)	7 (4 to 10)	0.041
Ventilator-free days	17.5 (0 to 23.3)	21 (0 to 25)	0 (0 to 21)	<0.001
ICU support				
Renal replacement therapy	69 (21.7)	54 (25.8)	15 (13.3)	0.008
Vasopressors	247 (77.4)	162 (77.5)	85 (75.2)	0.629
Outcomes				
ICU length of stay (days)	11 (6 to 19)	11 (6 to 19)	11 (6 to 18)	0.633
Hospital length of stay (days)	20 (12 to 34)	21 (13 to 35.8)	19 (10 to 27)	0.084
ICU mortality	98 (30.4)	56 (26.8)	42 (37.2)	0.054
Hospital mortality	125 (38.8)	73 (34.9)	52 (46)	0.051

Data presented as median (25 to 75% interquartile range) or *n* (%). ARDS, acute respiratory distress syndrome; PO_2_/FiO_2_, arterial partial pressure of oxygen/fraction of inspired oxygen;. ^a^*P* value of the comparison between light and deep sedation.

In a multivariate analysis, age, Charlson Comorbidity Index >2, SAPS3, severe ARDS and deep sedation were independently associated with increased hospital mortality (Table 4).

**Table 4:** Factors associated with hospital mortality in a multivariate analysis

**Parameter**	**Odds ratio**	**95% confidence interval**	***P *** **value**
Age	1.02	1.00 to 1.03	0.047
Charlson comorbidity index >2	2.06	1.44 to 2.94	<0.001
SAPS3	1.02	1.00 to 1.04	0.038
Severe ARDS	1.44	1.09 to 1.91	0.011
Deep sedation	2.36	1.31 to 4.25	0.004

Area under receiver operating curve for predicted mortality (95% confidence interval) = 0.768 (0.718 to 0.813), *P* <0.001. Hosmer–Lemeshow χ^2^ = 6.5670, *P* = 0.5840. ARDS, acute respiratory distress syndrome; SAPS3, Simplified Acute Physiology Score 3.

## Discussion

In the present multicenter cohort study we evaluated the association of early sedation strategies with outcomes of adult mechanically ventilated patients in the ICU. Deep sedation in the first 48 hours of MV is a common characteristic, observed in 35.1% of patients. Deep sedation was associated with increased disease severity, longer duration of ventilatory support and higher need for tracheostomy. In a multivariate analysis, deep sedation was an independent factor associated with increased hospital mortality (odds ratio = 2.36).

The more frequently used sedative regimens in our study were the association of midazolam and fentanyl or of propofol and fentanyl. When compared by sedation depth, the administration of fentanyl or dexmedetomidine as single drugs was more frequent in lightly sedated patients (15.3% vs. 7.1% for fentanyl, *P* = 0.033; 8.6% vs. 0.9% for dexmedetomidine, *P* = 0.005). These findings may be attributed to a rapid onset and offset of action of these drugs and their easiness to titrate, while benzodiazepines are more likely to accumulate, especially during continuous infusion and when associated with other drugs [26,27]. Recent studies demonstrate that sedation with benzodiazepines aiming at the same target is usually associated with a higher rate of sedation depth beyond the target as compared with dexmedetomidine [28].

Although important studies were published recently, a systematic review published in 2010 showed that clinical trials on sedation are commonly reported as low quality, mainly due to their design (for example, before and after studies), heterogeneous protocols and the relatively small number of patients in a single center or few centers [29]. These factors generate limited evidence to guide sedation drug choice and administration strategies in mechanically ventilated patients. Moreover, most studies – including recent randomized clinical trials – have enrolled patients 24 to 48 hours after the initiation of MV, making the clinical relevance of sedation depth in the early period more difficult to understand. A recent multicenter prospective cohort study by Shehabi and colleagues enrolled 251 patients, aiming to characterize the patterns of early sedation practice in 25 Australia and New Zealand (ANZ) ICUs and to assess its relationship with relevant clinical outcomes. After adjusting for potential confounding, early deep sedation was a predictor of time to extubation and mortality [30]. These results were corroborated by a cohort study in 11 Malaysian hospitals including medical/surgical patients (*n* = 259) who were sedated and ventilated ≥24 hours [31]. Likewise, in our study we could assess sedation strategies in the first 48 hours of MV and its relevant clinical outcomes in a large number of patients (*n* = 322), and demonstrated that early deep sedation was an independent predictor of hospital mortality.

Unlike the other variables that were also found to be independently associated with mortality in our study, such as ARDS severity, comorbidities and disease severity, sedation depth is a potentially modifiable risk factor for mortality. Implementing sedation protocols to achieve light sedation has been proven feasible and reproducible [32,33]. This evidence is important because deep sedation still seems to be current practice in many ICUs worldwide [5,13] and is present in 35% of the patients enrolled in the present study. Accordingly, Payen and colleagues observed in their survey a large proportion of patients in a deep state of sedation, and in addition no major changes in sedation depth or sedative dosages occurs during the first week of the ICU stay [34].

Finally, our findings provide validation of the concept that early sedation has a major impact on outcomes. Demonstrating this in a middle-income country in South America is an important step to provide generalizability of previous findings because the main previously published results on this issue are from ANZ ICUs. Several ANZ trials are well known for frequently presenting different results compared with similar interventions in other parts of the world, as demonstrated by studies in the last decade in the critical care field [35,36] and even in the sedation area [37]. This discrepancy is attributed to substantial differences due to the high standards in process of care and staffing patterns, and is consequently translated into lower mortality in ANZ ICUs [38,39].

The present study has some limitations that must be considered. First, the sedation level was not assessed using a specific sedation scale. Nonetheless, although originally created to assess the level of consciousness after head injury, it is known that GCS (the chosen scale) <9 denotes a comatose state even in nontraumatic settings, such as cerebral depression by pharmacological cause [40,41]. Moreover, the GCS shows excellent correlation and discrimination with the RASS (*r* = 0.91, *P* < 0.001), as described by Ely and colleagues from 1,360 paired observations among 275 adult patients in medical and coronary ICUs [25]. This strong correlation between the RASS and the GCS was also shown in other sedation scale validation studies [42,43], which allows its applicability as a surrogate for sedation depth in the present study.

Another limitation is the potential influence of previous neurological status on GCS values. Data regarding GCS on day 1 were collected in patients who were already sedated and under MV. A reported low GCS could therefore be influenced by the baseline neurological status and not only by sedation strategies, because it may also reflect acute neurological conditions associated with the current illness presented by the critically ill, such as encephalopathy. However, this is not an exclusive limitation of the GCS because this may also occur with the more specific sedation assessment scales such as the RASS or Sedation-Agitation Scale (SAS). Moreover, it is possible that certain patients may have required larger sedative doses on day 2 of MV for other reasons (ICU procedures, for instance) and we did not collect these data. In order to minimize the impact of other conditions in GCS values, we excluded patients with primary or known acute neurological disorders and included only patients under sedation in the present study. Considering the potential confounders mentioned above, the strong association between deep sedation and mortality must be analyzed regarding our interpretation of GCS values mainly as a surrogate of sedation depth.

Finally, we have not evaluated the presence of delirium. In mechanically ventilated patients, delirium is an independent predictor of hospital and 6-month mortality [6,44]. These outcomes are shown even in light sedated patients, where a dose–effect response was described [45]. Despite this clinical relevance, the incidence of delirium could not be evaluated in our study, since it was not the aim of the primary analysis. Finally, participant ICUs reported the existence of a sedation protocol, but the actual adherence for local recommendations was not assessed in the present study as it was beyond its initial scope.

## Conclusions

Early deep sedation is associated with worse outcomes and constitutes an independent predictor of hospital mortality in a prospective cohort of mechanically ventilated patients. Randomized clinical trials should be designed to address the impact of light versus deep sedation in the early phase (<48 hours) of MV.

## Key messages

• Early deep sedation is associated with longer duration of ventilator support and higher need for tracheostomy in mechanically ventilated patients.

• Early sedation depth is independently associated with increased hospital mortality.

• Sedation depth is a potentially modifiable risk factor for adverse outcomes; future clinical trials of light sedation with early (<24 hours) randomization of patients should therefore be encouraged.

## Abbreviations

ANZ: Australia and New Zealand; ARDS: acute respiratory distress syndrome; GCS: Glasgow Coma Scale; MV: mechanical ventilation; RASS: Richmond Agitation–Sedation Scale; SAPS3: Simplified Acute Physiology Score 3; SOFA: Sequential Organ Failure Assessment.

## Competing interests

This study was performed with institutional funds. JIFS received honoraria and unrestricted research grants from Hospira, Inc. São Paulo, Brazil, but this did not interfere with the design, analysis or the elaboration of the manuscript and therefore with adherence to the ethical requirements of the journal. The remaining authors declare that they have no competing interests.

## Authors’ contributions

LMST participated in study conception, data acquisition, data analysis and interpretation, and drafting of the manuscript. LCPA participated in study conception, data acquisition, data analysis and interpretation, and drafting of the manuscript. MP participated in study conception, data analysis and revising the manuscript for important intellectual content. GS participated in study conception, data acquisition, data analysis and drafting of the manuscript. APNJr participated in data acquisition, drafting of the manuscript and revising the manuscript for important intellectual content. AR-N participated in data acquisition and revising the manuscript for important intellectual content. LT participated in data acquisition and revising the manuscript for important intellectual content. VCdS-D participated in data acquisition and revising the manuscript for important intellectual content. AT participated in data acquisition and revising the manuscript for important intellectual content. TL participated in data acquisition and revising the manuscript for important intellectual content. CP participated in data acquisition and revising the manuscript for important intellectual content. FBC participated in data acquisition and revising the manuscript for important intellectual content. MdOM participated in data acquisition and revising the manuscript for important intellectual content. FPG participated in data acquisition and revising the manuscript for important intellectual content. FRM participated in data acquisition and revising the manuscript for important intellectual content. FD-P participated in data acquisition and revising the manuscript for important intellectual content. AGRdC participated in data acquisition and revising the manuscript for important intellectual content. RBdS participated in data acquisition and revising the manuscript for important intellectual content. PFGMMT participated in data acquisition and revising the manuscript for important intellectual content. MS participated in study conception, data analysis and interpretation, and drafting of the manuscript. JIFS participated in study conception, data analysis and interpretation, and drafting of the manuscript. All authors approved the final copy of the manuscript.

## Supplementary Material

Additional file 1 Is a table listing the sites, investigators and institutional review boards that participated in the Epidemiology of Respiratory Insufficiency in Critical Care study. Click here for file
