# Supplementary material for: Early sedation and clinical outcomes of mechanically ventilated patients: a prospective multicenter cohort study
Source: Crit Care. 2014 Jul 21;18(4):R156. doi: 10.1186/cc13995 (PMC4223597; doi:10.1186/cc13995)
Supplement: Additional file 1 — Is a table listing the sites, investigators and institutional review boards that participated in the Epidemiology of Respiratory Insufficiency in Critical Care study. [file cc13995-S1.docx]

Additional file 1: Participating ICUs, investigators and IRBs by state.

| **State** | **City** | **Participating ICU** | **Investigators** | **Institutional Review Board** |
| --- | --- | --- | --- | --- |
| Bahia | Salvador | Hospital Espanhol | Amadeu Martinez, Lívia Leal, Antonio Jorge Pereira | Comitê de Ética em Pesquisa do Hospital Espanhol |
| Distrito Federal | Brasília | Hospital Santa Luzia | Marcelo de Oliveira Maia, José Aires Neto | Comite de Ética em Pesquisa - Hospital Sirio-Libanes |
| Espírito Santo | Vitória | Vitória Apart Hospital | Claudio Piras | Comitê de Ética em Pesquisa do Vitória Apart Hospital |
|  | Vitória | Centro Integrado de Atenção à Saude (CIAS) Unimed Vitória | Eliana Bernadete Caser, Cora Lavigne Moreira | Comitê de Ética em Pesquisa em Seres Humanos – Hospital Unimed Vitória |
|  | Cariacica | Hospital Meridional | Pablo Braga Gusman, Dyanne Moysés Dalcomune | Comite de Etica em Pesquisa - Hospital Sirio-Libanes |
| Maranhão | São Luis | UDI Hospital | Alexandre Guilherme Ribeiro de Carvalho, Louise Aline Romão Gondim, Lívia Mariane Castelo Branco Reis | Comite de etica em Pesquisa - Hospital Sirio-Libanes |
| Minas Gerais | Belo Horizonte | Hospital Madre Tereza | Daniel da Cunha Ribeiro, Leonardo de Assis Simões, Rafaela Siqueira Campos, José Carlos Fernandez Versiani dos Anjos | Comitê de Ética em Pesquisa do Hospital Madre Teresa |
|  |  | Hospital Mater Dei | Frederico Bruzzi Carvalho | Comitê de Ética em Pesquisa do Hospital Mater Dei |
| Pará | Redenção | Hospital Regional Público do Araguaia | Rossine Ambrosio Alves, Lilian Batista Nunes | Comitê de Ética em Pesquisa da Fundação de Medicina Tropical do Tocantins |
| Paraná | Curitiba | Hospital do Trabalhador | Álvaro Réa-Neto, Mirella Cristine de Oliveira | Comitê de Ética em Pesquisa do Hospital do Trabalhador |
|  |  | Hospital Vita Batel | Álvaro Réa-Neto, Mirella Cristine de Oliveira | Comitê de Ética em Pesquisa do Hospital do Trabalhador |
|  |  | Hospital Universitário Cajuru | Álvaro Réa-Neto, Luana Tannous | Comitê de Ética em Pesquisa do Hospital do Trabalhador |
|  |  | Instituto de Neurologia de Curitiba (INC) | Álvaro Réa-Neto, Brenno Cardoso Gomes | Comitê de Ética em Pesquisa do Hospital do Trabalhador |
| Rio de Janeiro | Rio de Janeiro | Instituto Nacional de Câncer - Hospital do Câncer I | Vicente Cés de Souza Dantas | Comite de ética em pesquisa em seres humanos - INCA |
|  |  | Instituto de Pesquisa Clínica Evandro Chagas (IPEC) | Andre Japiassu | Comite de Ética em Pesquisa - Instituto de Pesquisa Clínica Evandro Chagas - Fundação Oswaldo Cruz |
|  |  | Hospital São Lucas | Marcos Freitas Knibel, Micheli Ponte, Pedro Mendes de Azambuja Rodrigues | Comite de Ética em Pesquisa - Hospital pró-cardiaco |
|  |  | Hospital Pasteur | Pedro Varaschin, Vicente Cés de Souza Dantas | Comite de Ética em Pesquisa - Hospital pró-cardiaco |
|  |  | Hospital da Mulher Heloneida Studart | Hélder Konrad de Melo, Elton Afonso Lopes | Comitê de Etica em Pesquisa do Hospital da Mulher Heloneida Studart |
|  |  | Hospital Pró-Cardíaco | Rubens Carmo Costa Filho, Felipe Saddy, Théia Forny Wanderley Castellões, Suzana Alves Silva | Comite de Ética em Pesquisa - Hospital pró-cardiaco |
|  |  | Nortecor Hospital de Clínica | Luiz Antonio Gomes Osorio, Dora Mannarino | Comite de etica em Pesquisa - Hospital Sirio-Libanes |
|  |  | Hospital Copa D'Or | Rodolfo Espinoza, Cassia Righy, Marcio Soares, Jorge Salluh, Lilian Tanaka, Daniel Aragão, Maria Eduarda Tavares, Maura Goncalves Pereira Kehdi | Comite de Ética em Pesquisa - Hospital Copa D'Or |
|  | Niterói | Hospital de Clínicas de Niterói | Fernando Borges Rodriguez, Priscila Abelha | Comite de Etica em Pesquisa - Hospital das Clinicas de Niterói |
|  | Duque de Caxias | Hospital de Clínicas Mario Lioni | Marcelo E. Lugarinho | Comite de etica em pesquisa - Hospital Mario Lioni |
| Roraima | Boa Vista | Hospital Geral de Roraima | Valéria Maria Campos Rezende, Roberto Carlos Cruz Carbonell | Comite de Ética em Pesquisa - Hospital Sirio-Libanes |
| Rio Grande do Sul | Porto Alegre | Hospital Moinhos de Vento | Cassiano Teixeira, Roselaine Pinheiro de Oliveira, Juçara Gasparetto Maccari, Priscylla Souza Castro | Comite de Ética em Pesquisa - Hospital Moinhos de Vento |
|  |  | Santa Casa de Misericórdia de Porto Alegre - Pavilhão Pereira Filho | Paula Berto, Patricia Schwarz | Comitê de Ética em Pesquisa - Irmandade Santa Casa de Misericordia de Porto Alegre |
|  |  | Santa Casa de Misericórdia de Porto Alegre - Hospital Santa Rita | André Peretti Torelly, Thiago Lisboa, Paula Berto, Edison Moraes | Comitê de Ética em Pesquisa - Irmandade Santa Casa de Misericordia de Porto Alegre |
| Santa Catarina | Criciúma | Hospital São José | Felipe Dal-Pizzol, Cristiane Tomasi Damiani, Cristiane Ritter | Comite de etica do Hospital São Jose. |
| São Paulo | Barretos | Fundação Pio XII - Hospital de Câncer de Barretos | Cristina Prata Amendola, Amanda Maria R. R. de Oliveira, Ulysses V. A. Silva, Luciana Coelho Sanches, Rosana D. S. Almeida | Comitê de Ética em Pesquisa da Fundação Pio XII - Hospital de Câncer de Barretos |
|  | Américo Brasiliense | Hospital Estadual de Américo Brasiliense | Paula Menezes Luciano, Evelin Drociunas Pacheco | Comite de ética em Pesquisa - Hospital das Clinicas da Faculdade de Medicina de Ribeirão Preto - USP |
|  | São Paulo | A. C. Camargo Cancer Center | Juliana Carvalho Ferreira, Ramon Teixeira Costa, Pedro Caruso | Comitê de Ética em Pesquisa - CEP - do A.C.Camargo Cancer Center |
|  |  | Hospital Sírio Libanês | Luciano Cesar Azevedo, Marcelo Park, Guilherme Schettino | Comitê de Ética em Pesquisa - Hospital Sírio-Libanês |
|  |  | Hospital Israelita Albert Einstein | Murillo Santucci Assunção, Eliezer Silva | Comite de Ética em Pesquisa - Hospital Israelita Albert Einstein |
|  |  | Hospital São Camilo Santana | Carlos Eduardo Barboza, Antonio Paulo Nassar Junior | Comitê de Ética em Pesquisa - Hospital São Camilo |
|  |  | Hospital São Camilo Pompéia | Antonio Paulo Nassar Junior | Comitê de Ética em Pesquisa - Hospital São Camilo |
|  |  | Hospital das Clinicas da Faculdade de Medicina da USP - UTI Disciplina Emergências Clinicas | Luciano Cesar Azevedo, Marcelo Park | Comissão de Ética para Análise de Projetos de Pesquisa - HCFMUSP |
|  |  | Hospital das Clinicas da Faculdade de Medicina da USP - UTI Disciplina Emergências Cirúrgicas | Paulo Fernando Guimarães Morando Marzocchi Tierno, Luis Marcelo Malbouisson, Lucas Oliveira | Comissão de Ética para Análise de Projetos de Pesquisa - HCFMUSP |
|  |  | Hospital das Clinicas da Faculdade de Medicina da USP - UTI Disciplina Anestesiologia | Davi Cristóvão | Comissão de Ética para Análise de Projetos de Pesquisa - HCFMUSP |
|  |  | Hospital Ipiranga - Rede Amil | Manoel Leitão Neto, Ênio Rego, Fernanda Eugênia Fernandes | Comite de Ética em Pesquisa - Hospital Sirio-Libanes |
|  |  | Hospital Do Coração | Marcelo Luz Pereira Romano, Alexandre Biasi Cavalcanti, Dalton de Souza Barros, Érica Aranha Suzumura, Karla Loureiro Meira, Gustavo Affonso de Oliveira | Comitê de Ética em Pesquisa - Hcor |
|  |  | Hospital São Paulo da Universidade Federal de São Paulo | Bruno Franco Mazza, Flavia Ribeiro Machado, Elaine Ferreira | Comite de Ética em Pesquisa - UNIFESP/EPM |
|  |  | Hospital Universitário da Universidade de São Paulo | Ronaldo Batista dos Santos, Alexandra Siqueira Colombo, Antonio Carlos Nogueira, Juliana Baroni Fernandes, Raquel Siqueira Nóbrega, Barbara do C.S. Martins, Francisco Soriano | Comitê de Ética em Pesquisa do Hospital Universitário da Universidade de São Paulo |
|  |  | Hospital São Luiz Jardim Anália Franco | Rafaela Deczka Morsch, Andre Luiz Baptiston Nunes | Comitê de Ética em Pesquisa - Beneficiência Médica Brasileira S/A Hospital São Luiz |
|  |  | Instituto do Câncer do Estado de São Paulo (ICESP) | Juliano Pinheiro de Almeida, Ludhmila Hajjar, Sílvia Moulin | Comitê de Ética em Pesquisa - FMUSP |
|  |  | Hospital e Maternidade São Luiz - Unidade Itaim | Fábio Poianas Giannini, Andre Luiz Baptiston Nunes | Comitê de Ética em Pesquisa - Beneficiência Médica Brasileira S/A Hospital São Luiz |
